# Novel high-resolution computed tomography-based radiomic classifier for screen-identified pulmonary nodules in the National Lung Screening Trial

**DOI:** 10.1371/journal.pone.0196910

**Published:** 2018-05-14

**Authors:** Tobias Peikert, Fenghai Duan, Srinivasan Rajagopalan, Ronald A. Karwoski, Ryan Clay, Richard A. Robb, Ziling Qin, JoRean Sicks, Brian J. Bartholmai, Fabien Maldonado

**Affiliations:** 1 Division of Pulmonary and Critical Care Medicine, Mayo Clinic, Rochester, MN, United States of America; 2 Department of Biostatistics and Center for Statistical Sciences, Brown University School of Public Health, Providence, Rhode Island, United States of America; 3 Department of Physiology and Biomedical Engineering, Mayo Clinic, Rochester, MN, United States of America; 4 Center for Statistical Sciences, Brown University School of Public Health, Providence, Rhode Island, United States of America; 5 Department of Radiology, Mayo Clinic, Rochester, MN, United States of America; 6 Division of Allergy, Pulmonary and Critical Care Medicine, Vanderbilt University, Nashville, TN, United States of America; Centro per lo Studio e la Prevenzione Oncologica, ITALY

## Abstract

**Purpose:**

Optimization of the clinical management of screen-detected lung nodules is needed to avoid unnecessary diagnostic interventions. Herein we demonstrate the potential value of a novel radiomics-based approach for the classification of screen-detected indeterminate nodules.

**Material and methods:**

Independent quantitative variables assessing various radiologic nodule features such as sphericity, flatness, elongation, spiculation, lobulation and curvature were developed from the NLST dataset using 726 indeterminate nodules (all ≥ 7 mm, benign, n = 318 and malignant, n = 408). Multivariate analysis was performed using least absolute shrinkage and selection operator (LASSO) method for variable selection and regularization in order to enhance the prediction accuracy and interpretability of the multivariate model. The bootstrapping method was then applied for the internal validation and the optimism-corrected AUC was reported for the final model.

**Results:**

Eight of the originally considered 57 quantitative radiologic features were selected by LASSO multivariate modeling. These 8 features include variables capturing Location: vertical location (Offset carina centroid z), Size: volume estimate (Minimum enclosing brick), Shape: flatness, Density: texture analysis (Score Indicative of Lesion/Lung Aggression/Abnormality (SILA) texture), and surface characteristics: surface complexity (Maximum shape index and Average shape index), and estimates of surface curvature (Average positive mean curvature and Minimum mean curvature), all with P<0.01. The optimism-corrected AUC for these 8 features is 0.939.

**Conclusions:**

Our novel radiomic LDCT-based approach for indeterminate screen-detected nodule characterization appears extremely promising however independent external validation is needed.

## Introduction

With approximately 160,000 deaths annually in the US, lung cancer continues to account for more cancer-related deaths than colon, prostate and breast cancer combined.[1] In 2011, the National Lung Screening Trial (NLST) demonstrated a 20% relative reduction in lung cancer mortality with annual low-dose computed tomography (LDCT).[2] These encouraging results triggered the widespread endorsement of lung cancer screening. However large-scale implementation has been hampered by the high rate of false-positive LDCT studies.[3] In the NLST approximately 40% of individuals randomized to LDCT screening had one or more pulmonary nodules identified during the study period, 96% of which were ultimately proven benign. [2]

In addition to lung cancer screening the increasing utilization of diagnostic chest computed tomography (CT) results in an estimated 1.5 million incidentally discovered indeterminate lung nodules in the US annually. With the implementation of LDCT lung cancer screening for the > 10 million US adults meeting the screening eligibility criteria, this number is estimated to increase substantially.[4]

In summary there appears to be a potential emerging global epidemic of newly detected lung nodules.[5] This increased detection of indeterminate pulmonary nodules in the absence of reliable non-invasive strategies to differentiate benign and malignant nodules will almost certainly result in an increase in iatrogenic mortality, treatment related morbidity and health care costs. While unnecessary invasive diagnostic and therapeutic interventions were kept to a minimum in the NLST study, the management of indeterminate pulmonary nodules in clinical practice serving the general population remains a major challenge.[2] Clinical risk calculators have significantly improved the management of indeterminate pulmonary nodules, but additional tools to distinguish benign from malignant nodules are needed, especially for intermediate risk pulmonary nodules, in order to minimize patient anxiety, radiation exposure, health care costs, and procedural morbidity and mortality.[6–11]

We have previously demonstrated that quantitative volumetric CT-based nodule characterization effectively risk-stratifies lung nodules of the adenocarcinoma spectrum.[12–16] In addition we have recently reported in a Lung Tissue Research Consortium based case control study that radiological features of the nodule surrounding lung tissue are potentially valuable in distinguishing benign from malignant lung nodules. (manuscript submitted)

This approach eliminates the intra- and inter-observer variability and is independent of the training level of the interpreting radiologist. In addition, modern digital CT images include a large amount of valuable high-dimensional data that currently is not fully utilized besides contributing to the overall impression “gestalt” by the radiologist. This invaluable resource can be leveraged by modern quantitative imaging methods. Radiomic approaches to lung nodule analysis consist of extracting reproducible and objective quantitative radiological variables from CT datasets, reducing large volumes of complex data to manageable and clinically relevant information.[17] These quantitative imaging techniques have been proposed to facilitate the development of diagnostic and prognostic models in lung imaging, allowing for example the risk-stratification of lung adenocarcinomas, the classification of screen- or incidentally detected lung nodules and the characterization of lung cancer subtypes and tumor heterogeneity.[14, 18–23] In this study, we used the NLST dataset to develop and internally validate a radiological multivariate model to distinguish malignant from benign CT-screen detected indeterminate pulmonary nodules.

## Methods

### Subject selection

The Mayo Clinic and Vanderbilt University Institutional Review Boards approved or exempted this study (IRB numbers: Vanderbilt University 151500 and Mayo Clinic 15–002674). All participants for the present study were selected from the pool of eligible participants in the NLST, and all patient data were fully anonymized. The methods of the NLST have been published elsewhere.[2, 24] Briefly, the NLST was a randomized controlled trial conducted at 33 US centers, approved by the Institutional review boards at all centers. The study recruited asymptomatic high-risk individuals from August 2002 through April 2004, aged 55 to 74 years, with a smoking history of at least 30 pack-years, who quit 15 years or less prior to randomization. Individuals were screened with either annual low-dose CT or chest X-ray for three years and followed through December 31, 2009. 26,722 individuals were randomized to the low-dose CT arm, and over 10,000 nodules (4–30 mm in longest diameter) were detected during the screening rounds.

Participants for the present study were selected from the pool of eligible participants in the NLST, who did not withdraw from follow-up, in the CT arm of the study (N = 26,262) and included all screen-detected lung cancer cases: adenocarcinomas, squamous cell carcinomas, large cell carcinomas, small cell carcinomas and carcinoid tumors. Non-lung cancer controls were selected as a stratified random sample from all participants without a diagnosis of lung cancer during the screen or follow-up periods of the NLST. Cases with more than one nodule were excluded. We restricted our analysis to pulmonary nodules with a size defined by a largest diameter between 7 and 30 mm as reported in the NLST database.

### Screening HRCT data

All NLST screening scans were low-dose scans with 2.5 mm collimation or less as pre-defined by strict NLST criteria, the details of which have been published elsewhere.[24] The CT datasets were obtained from the Lung Screening Study core laboratory and transferred to a hard drive that was shipped to the investigators. The datasets from the American College of Radiology Imaging Network core laboratory were transferred initially via hard drive, then electronically to the investigators. Information on nodule location was available to the investigators in the NLST database and confirmed by one radiologist (B.J.B.) and two pulmonologists (F.M. and T.P.) using the CT obtained closest in time to the diagnosis of malignant or benign lung nodules. Nodules were electronically tagged for segmentation and analysis. HRCT without visible nodules, nodules with borders indistinguishable from neighboring structures (e.g. mediastinum or pleura) and nodules without related clinical data were excluded.

### Optimization and validation of nodule segmentation

The lung nodules were segmented manually using the ANALYZE software (Biomedical Imaging Resource, Mayo Clinic, Rochester, MN). The location and the extent of each nodule was identified visually and a stack of two dimensional borders were traced out along the transverse orientation. A semi-automated region-growing approach based on the operator-specified bounding cube enclosing the nodule and a seed location within the nodule was used for initial segmentation (see supporting information). Manual editing was performed to remove, if needed, intruding structures like vessels and pleura. A parametric feature-based region growing technique based on the texture classification of the voxels within the operator specified bounding cube was used as previously described.[25]

### Radiomic features

A comprehensive set of automatically computable, quantitative radiomic metrics was included for the development of a multivariable predictive model to discriminate benign from malignant lung nodules. Based on previous data and preliminary analysis (S1 File), we considered metrics within the following categories: general characteristics of the nodule (size and location), nodule characteristics (radiodensity, texture and surface characteristics) and features of the nodule-free surrounding lung characteristics, as below (Table 1):

1. Metrics capturing the spatial Location of the nodule.
2. Nodule Size
3. Bulk metrics based on the global Shape descriptors of the nodule.
4. Radiodensity metrics based on the CT Hounsfield units within the nodule.
5. Nodule Texture/Density metrics based on the texture exemplar distributions within the nodule.
6. Texture/Density nodule surrounding lung metrics based on the parenchymal texture exemplar distributions within a region surrounding the nodule.
7. Metrics capturing the nodule surface descriptors.
8. Metrics capturing the distribution of the nodule surface characteristics exemplars.

**Table 1:** AUC analysis across cancers and controls.

ID	Variables	Cancer mean (SD) n = 408	Control mean (SD) n = 318	AUC	P value
	**1. Location**				
20	Location	6.37 (3.42)	7.06 (3.16)	0.56	0.00558
1	Centroid_x	154.78 (74.5)	142.21 (78.73)	0.56	0.02837
2	Centroid_y	143.95 (47.18)	151.84 (55.47)	0.47	0.03916
3	Centroid_z	203.38 (60.1)	186.88 (65.91)	0.57	0.00052
	**2. Size**				
4	Volume*	3305.34 (6361.01)	345.45 (819.51)	0.90	2.55e-20
5	Surface Area*	1673.08 (2150.55)	345.04 (501.95)	0.87	4.45e-23
	**3. Shape**				
6	Sphericity	0.51 (0.21)	0.6 (0.29)	0.58	1.24e-05
7	Sphere Fit Factor	6.82 (8.31)	5.28 (5.82)	0.58	0.00724
8	Estimated Radius	7.61 (3.99)	3.59 (1.57)	0.90	5.34e-37
9	Minimum Enclosing Brick x	19.82 (12.12)	9.46 (5.51)	0.84	6.21e-29
10	Minimum Enclosing Brick y	19.63 (12.13)	10.11 (6.72)	0.82	3.11e-26
11	Minimum Enclosing Brick	16.49 (14.51)	4.97 (2.65)	0.92	1.24e-36
12	Maximum Brick length	24.08 (16.27)	11.31 (7.04)	0.84	6.69e-29
13	Elongation	-0.25 (0.4)	-0.31 (0.47)	0.57	0.0737
14	Flatness	-0.56 (0.99)	-1.01 (1.05)	0.66	7.33e-09
	**4. Radiodensity**				
15	HU_mean	-209.18 (163.55)	-465.23 (201.91)	0.83	1.52e-40
16	HU_variance	614546.92 (3444392.14)	295011.7 (609422.64)	0.56	0.0969
17	HU_skew	-2.64 (10.09)	-2.39 (1.2)	0.56	0.665
18	HU_kurtosis**	31.36 (91.51)	10.55 (10.05)	0.74	7.19e-12
19	HU_entropy	7.89 (1.77)	6.76 (1.76)	0.82	1.20e-22
	**5. Texture Nodule**				
21	SILA_Tex	122.91 (34.32)	58.62 (38.1)	0.88	2.56e-47
22	Texture_Risk	2.17 (0.57)	1.36 (0.54)	0.82	7.47e-42
	**6. Texture Nodule Surrounding Lung**				
23	Vessels	1.88 (2.8)	0.75 (1.29)	0.74	2.42e-13
24	Background	9.49 (9.56)	9.59 (11.25)	0.52	0.886
25	SILA_Fibrosis	32.32 (17.84)	27.42 (22.96)	0.57	0.00141
26	SILA_low attenuation	35.54 (6.33)	32.69 (19.86)	0.55	0.0363
	**7. Nodule Surface**				
27	Number of Vertices	2711.4 (4745.67)	515.25 (697.45)	0.88	4.97e-24
28	Number of Faces	5419.18 (9488.83)	1026.56 (1395.09)	0.88	5.05e-24
29	Willmore Bending Energy_2	1574.75 (3792.16)	480.61 (721.39)	0.75	1.27e-12
30	Willmore Bending Energy	2269.82 (6283.03)	802.67 (1116.04)	0.70	1.01e-09
31	Minimum Mean Curvature	-0.92 (0.65)	-0.28 (0.46)	0.82	4.37e-31
32	Maximum Mean Curvature	3.57 (2.44)	3.27 (1.82)	0.51	0.0731
33	Average Positive Mean Curvature	0.34 (0.11)	0.58 (0.2)	0.87	3.73e-39
34	Skew Positive Mean Curvature	2.89 (2.04)	2.01 (1.2)	0.66	2.15e-10
35	Minimum Gaussian Curvature	-1.01 (0.87)	-0.87 (0.84)	0.58	0.0381
36	Maximum Gaussian Curvature	15.43 (30.41)	12.6 (21.14)	0.52	0.172
37	Average positive Gaussian Curvature	0.29 (0.29)	0.61 (0.52)	0.79	7.37e-21
38	Skew Positive Gaussian Curvature	7.57 (3.82)	4.66 (2.09)	0.78	1.46e-24
39	Minimum Sharp	8.82e-05 (9.35e-04)	8.86e-04 (2.41e-03)	0.79	6.01e-07
40	Maximum Sharp	38.99 (62.98)	22.44 (52.57)	0.59	0.00028
41	Average Sharp	0.59 (0.43)	1.01 (0.78)	0.71	1.89e-15
42	Skew Sharp	7.95 (7.45)	4.25 (3.53)	0.72	3.16e-12
43	Minimum Curved	0.01 (0.03)	0.07 (0.1)	0.82	6.44e-18
44	Maximum Curved	5.72 (4.21)	4.8 (3.05)	0.53	0.00143
45	Average Curved	0.58 (0.19)	0.96 (0.32)	0.86	1.89e-38
46	Skew Curved	2.87 (2.26)	1.79 (1.25)	0.69	5.44e-12
47	Minimum Shape Index	-0.98 (0.01)	-0.98 (0.02)	0.63	9.85e-07
48	Maximum Shape Index	0.98 (0.16)	0.55 (0.61)	0.82	1.46e-17
49	Average Shape Index	-0.29 (0.18)	-0.55 (0.13)	0.88	5.51e-43
50	Skew Shape Index	1.63 (0.91)	1.72 (1.42)	0.54	0.306
51	Intrinsic Curvature Index	37.78 (118.81)	15.7 (21.56)	0.64	1.49e-06
52	Extrinsic Curvature Index	113.69 (284.16)	39.41 (57.05)	0.73	5.04e-11
	**8. Distribution of the nodule surface exemplars**				
53	SILA morpheme	36.02 (11.24)	19.71 (12.61)	0.84	5.21e-40
54	Morpheme Average Curvature	0.74 (0.23)	1.05 (0.32)	0.81	1.10e-29
55	Morpheme Skew Curvature	2.33 (1.73)	1.57 (1.04)	0.66	4.20e-10
56	Local SILA Average	27.65 (8.71)	15.3 (9.26)	0.84	5.08e-40
57	Local SILA Skew	0.71 (0.42)	0.49 (0.68)	0.60	1.46e-07

*: One case (ID 516) is the outlier and was removed from the calculations.

**: One case (ID 534) is the outlier and was removed from the calculations.

### Development of Score Indicative of Lesion/Lung Aggression/Abnormality (SILA)

Current literature suggests that no single quantitative metric exists to differentiate benign and malignant nodules. However, multivariate predictive models based on an ensemble of nodule texture/density, surround texture/density, nodule surface and other shape descriptors could improve the discriminability. To facilitate the multivariate analysis we investigated the possibility of replacing our previously developed nodule texture/density and surface categorization using unsupervised stratification into continuous variables that can be thresholded at multiple levels to provide, if needed, the necessary categorization. We developed SILA to map the nine nominal texture/surface exemplar distributions of the nodule onto a continuous scale. The nine nominal exemplar distributions can be ordinated in 362,880 (factorial 9) ways. To identify the unique ordination that correlates with the virulence/malignancy of the nodule, we used qualitative spatial reasoning and multi-dimensional scaling. Based on this, the nine texture exemplars arbitrarily labeled as V,I,B,G,Y,O,R,C, and P were ordinated as V-R-O-I-Y-P-B-G-C identical to that used to represent the distributions via the glyphs (Figure C in S1 File). The nine surface exemplars were ordinated as unknown-minimal surface-valley-flat-ridge-pit-saddle valley-saddle ridge-peak. SILA was computed as the Cramer-Von Mises Distance of the ordinated exemplar distributions. Using a similar strategy, the seven primal parenchymal exemplars (Normal, Ground Glass, Honeycombing, Reticular, {mild, moderate, severe} lower attenuation areas) were ordinated to compute the SILA for the parenchyma surrounding the nodule (Figure D in S1 File shows the operator dependent variations in the SILA mappings for the texture and surface characterization. The 95% C.I for the maximum nodule-specific SILA differences across the 3 operators was 0.217–0.271 and 0.236–0.276 respectively for the texture and surface characterization).

### Multivariate model

Quantitative methods were developed to characterize independent radiological variables assessing various radiologic nodule features. Univariate analysis of the discriminatory power of each radiologic variable and receiver operative curve (ROC) analysis were performed for each variable and an area under the curve (AUC) calculated. Statistical significance was calculated and adjusted for multiple comparisons using Bonferroni correction. Spearman rank correlations between all pairs of variables were calculated and displayed via a heat map. Multivariate analysis was performed using least absolute shrinkage and selection operator (LASSO) method for both variable selection and regularization in order to enhance the prediction accuracy and interpretability of the multivariate statistical model. To increase the stability of the modeling, LASSO was run 1,000 times and the variables that were selected by at least 50% of the runs were included into the final multivariate model.[26] The bootstrapping method was then applied for the internal validation, and the optimism-corrected AUC was reported for the final model.

## Results

### Study participants

**Fig 1:**
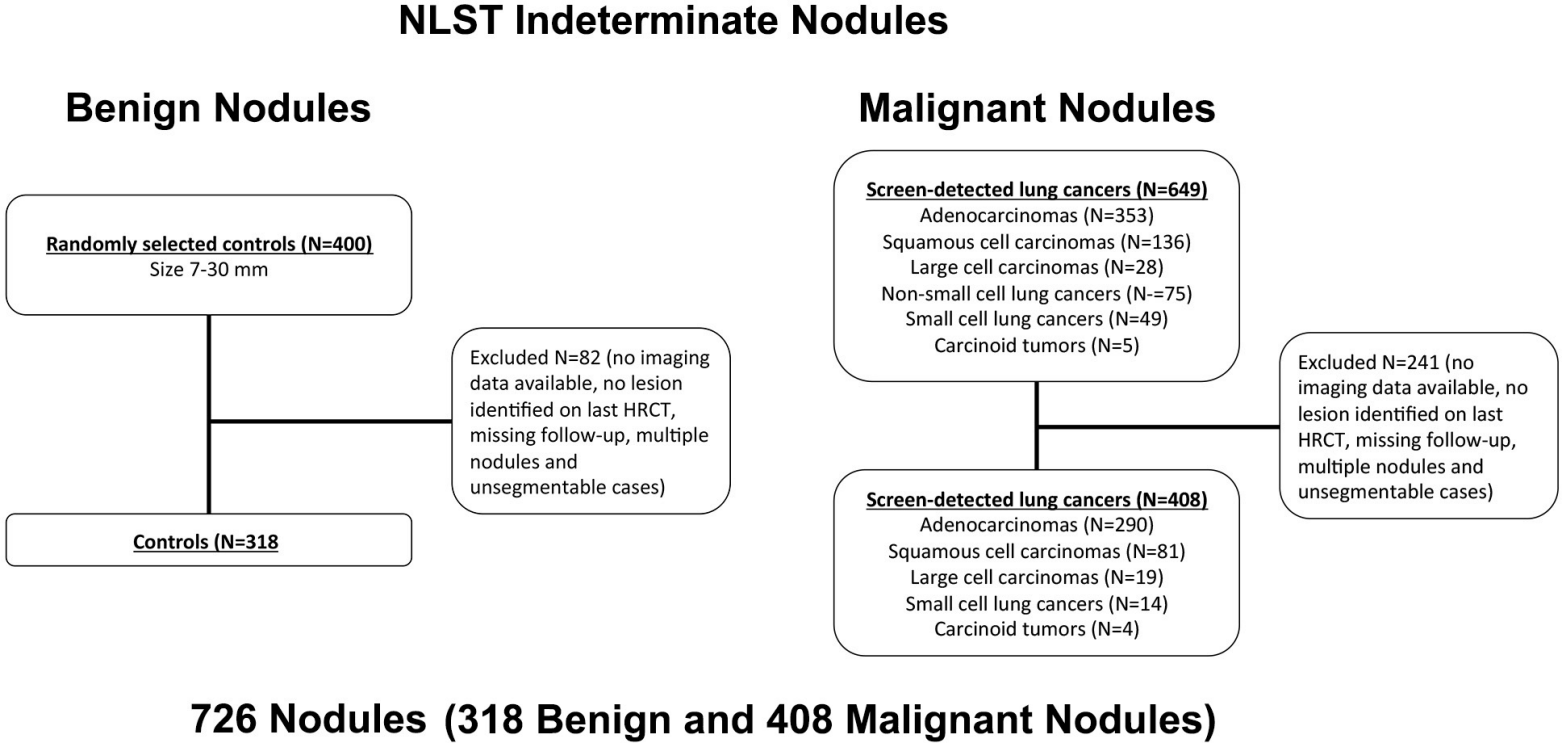
Flow chart of nodule selection.

We reviewed 649 LDCT of cancers diagnosed in the screening arm of the NLST that included 353 adenocarcinomas, 136 squamous cell carcinomas, 28 large cell carcinomas, 75 non-small cell carcinomas, 49 small cell carcinomas and 5 carcinoid tumors. After exclusion of cases lacking HRCT data, cases with no apparent lesion on last HRCT prior to the cancer diagnosis, cases with nodules invading the mediastinum, cases with missing outcome data, and lesion with size < 7mm or >30 mm, 408 LDCT scans with malignant nodules were selected and analyzed. A stratified random sample of non-lung cancer control nodules (size between 7 and 30 mm) was selected on a 1:1 basis, and 318 benign nodules were selected and included in the analysis (Fig 1). The demographic and clinical characteristics of individuals included in the study are summarized in Table 1.

In order to prevent overfitting of the model, we only considered quantitative imaging variables that were known *a priori* to be potentially associated with the benign or malignant nature of lung nodules (see supplemental material). Quantitative methods were developed to characterize independent radiological variables assessing various radiologic nodule features including 1. Nodule location, 2. Nodule size, 3. Nodule shape, 4. Nodule radiodensity 5. Nodule texture, 6. Texture/radiodensity of the nodule-free surrounding lung, 7. Nodule surface characteristics and 8. Distribution of the nodule surface characteristics exemplars using 726 nodules identified from the NLST dataset (benign, n = 318 and malignant, n = 408). (Table 2)

**Table 2:** Demographics and clinical characteristics of cancer and control (n = 726).

	Lung Cancer Cases (n = 408)	Nodule-Positive Controls (n = 318)	p Value
**Age, mean ± SD, y**	63.7 ± 5.3	61.2 ± 5.0	<0.001
**Sex, n (%)**			0.45
**Male**	230 (56.4)	189 (59.4)	
**Female**	178 (43.6)	129 (40.6)	
**Race, n (%)**			0.03
**White**	385 (94.4)	286 (89.9)	
**Black, Asian, other**	23 (5.6)	32 (10.1)	
**Ethnicity, n (%)**			0.31
**Hispanic or Latino**	405 (98.4)	313 (99.3)	
**Neither Hispanic nor Latino**	3 (1.6)	5 (0.7)	
**Smoking, n (%)**			0.37
**Current**	221 (54.2)	161 (50.6)	
**Former**	187 (45.8)	157 (49.4)	
**Pack-years smoked, mean ± SD**			
**Current smokers**	64.8 ± 25.8	55.5 ± 20.9	<0.001
**Former smokers**	66.7 ± 30.6	55.2 ± 26.9	<0.001
**Self-reported history of COPD, n (%)**			
**Yes**	43 (10.5)	18 (5.7)	0.02
**No**	365 (89.5)	300 (94.3)	
**FH of lung cancer, n (%)**			0.08*
**Yes**	113 (28.9)	69 (22.8)	
**No**	278 (71.1)	233 (77.2)	
**Missing**	n = 17	n = 16	
**Stage, n (%)**			—
**I**	298 (73.0)	—	
**II**	29 (7.1)	—	
**III**	55 (13.5)	—	
**IV**	20 (5.0)	—	
**Carcinoid, unknown**	6 (1.5)	—	
**Histologic subtype, n (%)**			—
**Adenocarcinoma**	290 (71.1)	—	
**Squamous cell carcinoma**	81 (19.9)	—	
**Other, NOS, unknown**	37 (9.1)	—	

P Values calculated using Fisher’s exact test for categorical variables, Student’s t test for continuous variables.

* P value for family history of lung cancer was calculated without missing data.

### Multivariate analysis

**Fig 2:**
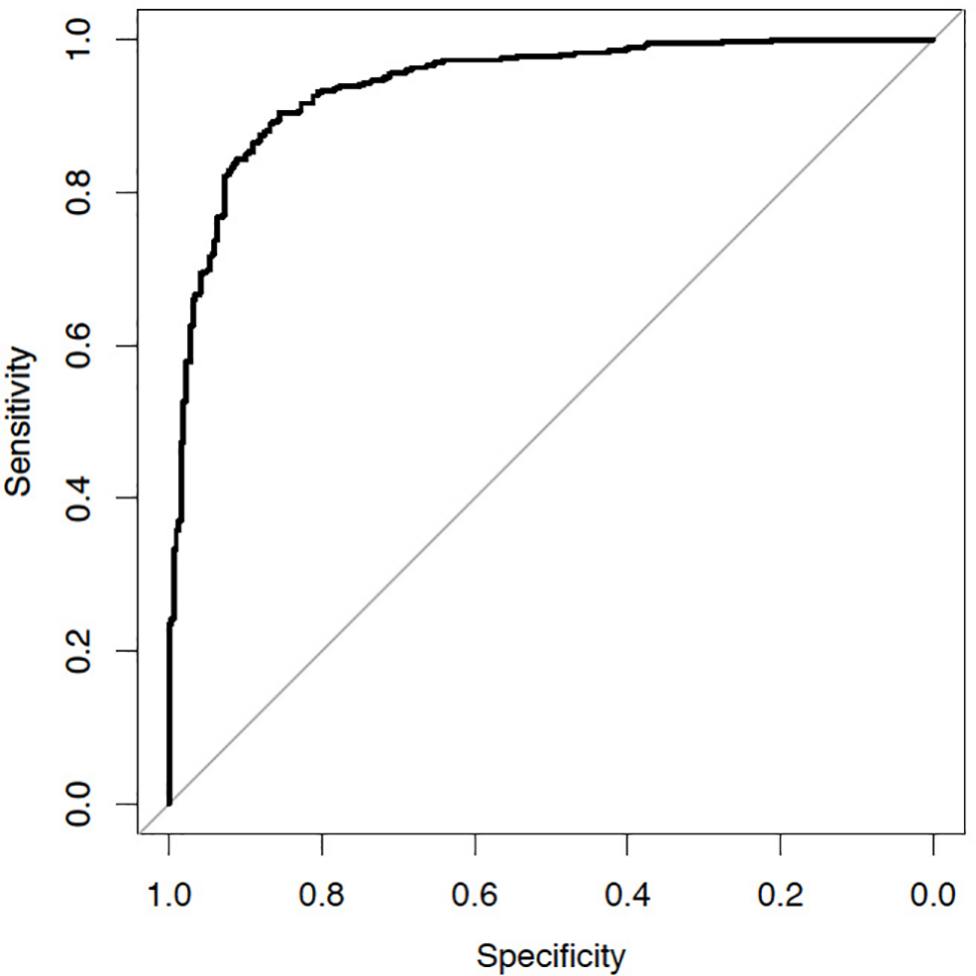
Receiver operating curve analysis.

In order to select the optimal variables, adjust the regression coefficients to optimize the transportability (external validity) of the model and determine the degree of optimism of the model and perform optimism-corrected analysis of the performance of the model by ROC analysis, all 57 quantitative imaging variables were included in the LASSO regression model. Multivariate analysis using LASSO on all features yielded a multivariate model with 8 selected features (selected with frequency > 50% after introducing bootstrap to reduce variability after 1000 runs) with an AUC estimate of 0.941. (Fig 2) These 8 features include: 1. Offset carina centroid_z (Nodule location), 2. Minimum enclosing brick (Nodule shape), 3. Nodule flatness (Nodule shape), 4. SILA nodule texture (Nodule texture), 5. Maximum shape index (Nodule surface Characteristics), 6. Average shape index (Nodule surface Characteristics), 7. Average positive mean curvature (Nodule surface Characteristics) and 8. Minimum mean curvature (Nodule surface Characteristics), all with P<0.01. To correct overfitting (internal validation) we used the bootstrapping technique to estimate the optimism of the AUC. The optimism-corrected AUC is 0.939 (Fig 2). Using Youdan's index, we obtained the optimal cutoff at 0.478 with sensitivity 0.904 and specificity 0.855. A subset analysis of nodules with size between 7 mm and 15 mm yielded an AUC of 0.9477 with an optimism-corrected AUC of 0.9409 (n = 169 nodules).

Offset carina centroid_z captures the location of the nodule in the vertical axis in relationship to the carina, the minimal enclosing brick and flatness capture shape and volume, SILA texture is a summary variable capturing the nodule texture, maximum and average shape index capturing the complexity of the nodule surface and average positive mean curvature and minimum mean curvature representing the degree of curvature of the outer surface of the nodule account for the surface characteristics of the nodule.

## Discussion

In this study, we report the development and the performance of an internally-validated multivariate radiomic model to differentiate malignant and benign screen-identified indeterminate lung nodules. Using a large lung cancer screening dataset of images obtained with a broad spectrum of CT scanners, acquisition protocols and reconstruction kernels, we demonstrate that our automated radiomic approach reliably distinguishes benign from malignant nodules. This approach, if externally validated, could inform management of screen-identified pulmonary nodules and potentially minimize morbidity, mortality, health care costs, radiation exposure and patient anxiety associated with the currently accepted approach for the evaluation and management of indeterminate pulmonary nodules.

To eliminate “agnostic” variables with unknown or improbable clinical significance we pre-selected quantitative imaging features with known potentially associations to the benign or malignant nature of lung nodules for our model. In addition to standard nodule descriptors such as size and location we include variables capturing nodule surface characteristics, density and characteristics of the nodule-free surrounding lung. Although a number of these additional features may influence the subjective assessment by trained radiologist, they currently cannot be accurately measured clinically.[27] While predictive in the univariate analysis features of the immediate nodule-free surrounding lung, as determined by quantitative estimates of low-attenuation (emphysema), groundglass and reticular changes within 10 mm of the segmented boundaries of the nodule were not found to be useful predictors after LASSO selection of candidate predictors. Interestingly, nodule size was not one of the eight selected variables. The only potential variable related to size was the minimum enclosing brick. In order to evaluate the performance of our model without the nodule size as a variable, the optimism-corrected AUC was calculated after removing each variable (Table 3). The AUC for the 7-variable model without minimum enclosing brick was 0.929, suggesting that nodule size did not exert a disproportionate influence on the final model.

**Table 3:** Model performance after removal of individual variables.

Removing Variable	Corrected AUC with optimism correction using bootstrapping	Difference from full model	Uncorrected AUC without bootstrapping	Difference from full model
Flatness	0.9394114	0.0001422	0.9405	-0.0005
SILA_Tex	0.9280794	-0.0111898	0.9294	-0.0116
Avg_PosMeanCurv	0.9399478	0.0006786	0.9411	0.0001
Max_SI	0.9387773	-0.0004919	0.9402	-0.0008
Avg_SI	0.9396861	0.0004169	0.9409	-1E-04
Centroid_Z	0.9366246	-0.0026446	0.9381	-0.0029
Min.Enclosing.Brick	0.9289959	-0.0102733	0.9312	-0.0098
Min_MeanCurv	0.9397763	0.0005071	0.9411	0.0001
All 8 variables	0.9392692		0.941	

If externally validated the excellent diagnostic test performance of our multivariate model could significantly advance the management of patients with screen-detected indeterminate pulmonary nodules. The development of this model based on a large and technically heterogeneous screening dataset including a geographically diverse population and various CT scanners and acquisition protocols, strengthen the external validity of our study.[2, 24] In addition, all analyzable nodules from the NLST were included in modeling which used model selection through shrinkage (LASSO) and bootstrap analysis, allowing adjustment for overfitting and validation of the modeling process.[26]

One the main limitations to broad implementation of lung cancer screening remains the large number of false positive screening CT. In order to mitigate this problem and decrease unnecessary patient complications, radiation exposure and patient anxiety, the nodule size threshold for a positive screening study was raised to 6 mm.[28–30] This size threshold has accordingly been endorsed by several other societies such as the Fleischner Society.[31] We selected a threshold of 7 mm in our study for its similarity with this threshold, and also for consistency with the DECAMP-1 study we are planning to use for external validation (NCT01785342). While this 6mm threshold is unquestionably an improvement over the NLST criteria, the number of false positive CT remains substantial, and this problem is likely to persist as screening is more broadly implemented and eligible individuals are screened over longer time periods. Another potentially fruitful avenue of research is the applications of longitudinal volumetric assessment of screen-identified lung nodules, which have been associated with a substantial reduction in the incidence of false-positive CT as well.[32, 33] In fact, the recent European position statement on lung cancer screening endorses volumetric analysis for lung nodule assessment.[34] While some blood or bronchoscopy-based biomarkers have been proposed to facilitate nodule classification, they require additional invasive procedures, which may be difficult to generalize at the population level.[25, 35–39] Leveraging existing and currently unexploited data to refine the sensitivity and specificity of LDCT would therefore be desirable. Our radiomics classifier compares favorably to currently existing clinical, blood or tissue or radiology-based prediction models and focuses specifically on lung nodule variables considered clinically relevant. Rather than replacing current clinical-based assessment of lung nodules based on size or volumetric analysis, we believe that our classifier could represent an adjunct diagnostic tool to inform clinical decisions for intermediate risk indeterminate pulmonary nodules. This radiomics approach would also not require additional expensive imaging such as PET-CT as required by other additive models.[7, 8, 10]

There are several limitations to our work. First, our model has not yet been externally validated before it is used clinically. The prevalence of malignancies in our cohort is > 50%, which is distinctly more than in a typical screening cohort including similar size lesions (12%). Consequently, it is unclear how our model will perform in independent screening cohorts with a more typical nodule prevalence. If our model cannot be validated it may have to be adjusted based on the validation cohort. However, we used an optimal internal validation model (LASSO), which not only surpasses conventional internal validation approaches (split sample and cross validation), but also penalizes the model to avoid overfitting and optimizes the generalizability of the model.

Second, the model was developed from a very heterogeneous sample of the NLST CT dataset and we found the selected radiomic features to be robust and stable across CT platforms, acquisition protocols and reconstructions kernels, which we believe strengthens the reproducibility of our model.

Third, the semi-automatic segmentation technique used in this study with manual adjustment by the investigators could admittedly introduce operator-driven variability in radiomic analysis. However, we have recently analyzed the reproducibility of radiomic analysis of adenocarcinomas using the same segmentation technique and found excellent Intraclass Correlation Coefficient (0.828 (95% CI 0.76, 0.895) for the Vanderbilt cohort of 50 adenocarcinomas.[40] We believe that these results support the external validity of our work.

Fourth, the relatively small number of cases did not allow us to exclude the influence of clinical or demographic variables known to affect lung cancer risk. We did, however, include additional clinical variables known to strongly influence the risk of lung cancer (age and smoking history in pack-years) and found that these variables did not improve the performance of the model. Finally, it is unclear whether our model will extend to other lung nodule cohorts, such as incidentally-detected lung nodules. Future validation of our model in other settings is indeed warranted.

Finally, it should be noted that all lung cancer cases suitable for analysis from the NLST were included in our study, some of which were at advanced stage (see Table 2). This could potentially limit the external validity of our model when applied to indeterminate pulmonary nodules. However most of the included cancer cases were stage I which should mitigate this risk.

In summary, we present a promising novel radiomics CT-based approach to lung nodule classification, which we believe could revolutionize our approach to screen-detected indeterminate pulmonary nodules and mitigate the risks inherent in lung cancer screening by minimizing unnecessary mortality, morbidity, radiation exposure, patient anxiety and healthcare costs.

## Supporting information

S1 File **Figure A.** Analysis of the CALIPER texture features within the lung nodules. The texture features within the shaded region do not appear within the lung nodules.
**Figure B** Three dimensional scatter plot showing the pairwise Dice Similarity Coefficient (DSC) between the nodules segmented by the Radiologist (Rx), Pulmonologist (Px) and Image Analyst (IA).
**Table A** Algorithmic components of nodule surface characterization and the strategy used during the pilot study and current improvements.
**Table B.** List of quantitative metrics used in the discrimination of benign and malignant nodules. The pval, 95% CI and the probability plot correlation coefficient (PPCC) are given in the last column for benign (N = 319) and malignant (N = 338) nodules.
**Figure C** Mosaic showing the glyphs (**A**, **D**), the nodule distribution within the upper, middle, lower left and right lung (**B**, **E**) and the Score Indicative of Lesion Abnormality (SILA) for the NLST malignant and benign nodules used in this study. The glyphs are ordered in Panels **A** and **D** based on the nodule-specific SILA values; the SILA values in Panels **C** and **F** are color coded in green, yellow and red based on the previously developed CANARY categorization.
**Figure D** Three dimensional scatter plot showing the variations in the SILA (Score Indicative of Lung Abnormality) between the nodules segmented by 3 operators. Panels A and B respectively show the SILA values for the nodule texture and surface. The nodules (N = 266) described in section 2.2.1 were used for this analysis.
(DOCX)
