# Correction: Novel high-resolution computed tomography-based radiomic classifier for screen-identified pulmonary nodules in the National Lung Screening Trial

**DOI:** 10.1371/journal.pone.0205311

**Published:** 2018-10-02

**Authors:** Tobias Peikert, Fenghai Duan, Srinivasan Rajagopalan, Ronald A. Karwoski, Ryan Clay, Richard A. Robb, Ziling Qin, JoRean Sicks, Brian J. Bartholmai, Fabien Maldonado

There are multiple errors in the Funding section. The correct funding information is as follows: This work was supported by the Office of the Assistant Secretary of Defense for Health Affairs, through the Lung Cancer Research Program under Award No. W81XWH-15-1-0110 (Fabien Maldonado). Opinions, interpretations, conclusions and recommendations are those of the author and are not necessarily endorsed by the Department of Defense. The funders had no role in study design, data collection and analysis, decision to publish, or preparation of the manuscript.
