# Supplementary material for: Novel high-resolution computed tomography-based radiomic classifier for screen-identified pulmonary nodules in the National Lung Screening Trial
Source: PLoS One. 2018 May 14;13(5):e0196910. doi: 10.1371/journal.pone.0196910 (PMC5951567; doi:10.1371/journal.pone.0196910)
Supplement: S1 File — Figure A. Analysis of the CALIPER texture features within the lung nodules. The texture features within the shaded region do not appear within the lung nodules. Figure B Three dimensional scatter plot showing the pairwise Dice Similarity Coefficient (DSC) between the nodules segmented by the Radiologist (Rx), Pulmonologist (Px) and Image Analyst (IA). Table A Algorithmic components of nodule surface characterization and the strategy used during the pilot study and current improvements. Table B. List of quantitative metrics used in the discrimination of benign and malignant nodules. The pval, 95% CI and the probability plot correlation coefficient (PPCC) are given in the last column for benign (N = 319) and malignant (N = 338) nodules. Figure C Mosaic showing the glyphs (A, D), the nodule distribution within the upper, middle, lower left and right lung (B, E) and the Score Indicative of Lesion Abnormality (SILA) for the NLST malignant and benign nodules used in this study. The glyphs are ordered in Panels A and D based on the nodule-specific SILA values; the SILA values in Panels C and F are color coded in green, yellow and red based on the previously developed CANARY categorization. Figure D Three dimensional scatter plot showing the variations in the SILA (Score Indicative of Lung Abnormality) between the nodules segmented by 3 operators. Panels A and B respectively show the SILA values for the nodule texture and surface. The nodules (N = 266) described in section 2.2.1 were used for this analysis. (DOCX) [file pone.0196910.s001.docx]

**1.1** **Optimization and validation of nodule segmentation.** During our initial feasibility study lung nodules were segmented manually using ANALYZE software (Biomedical Imaging Resource, Mayo Clinic, Rochester, MN). The location and the dimensions of each nodule were identified visually and a stack of two dimensional borders were traced out along the transverse orientation. Tracing errors were minimized by guiding the manual traces along the automatically detected edges. Apart from being very labor intensive and subjective, this manual approach suffers from out-of-plane discontinuities arising due to section-by-section two dimensional delineation of a three dimensional object. To optimize nodule segmentation, we developed a semi-automatic region growing approach based on the operator specified bounding cube enclosing the nodule and a seed location within the nodule. Manual editing tools were added to remove, if needed, intruding structures like vessels and pleura. Region growing approaches traditionally use the underlying voxel intensities to identify and agglomerate regions similar to the intensity at the seed location. Such an approach is highly sensitive to placement of the seed and the inherent noise in the scan. We developed a “parametric” feature-based region growing technique based on the texture classification of the voxels within the operator specified bounding cube. The texture classification is based on the Computer Aided Lung Informatics for Pathology Evaluation and Rating (CALIPER) software (Mayo Clinic, Rochester, MN). The detection and quantification of pulmonary parenchyma on CT scans by CALIPER is based on histogram signature mapping techniques trained through expert radiologist consensus assessment of pathologically confirmed training sets. Using CALIPER, we analyzed the signatures of a multitude of manually segmented nodules derived from the Mayo training, discovery and NLST lung cancer cohort (**Figure A**). Through this exercise, the candidate texture features for the parametric region growing were identified.


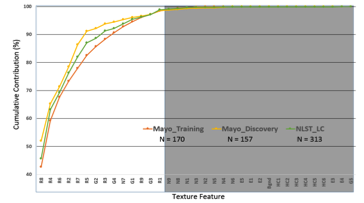


**Figure A.**

To validate the reproducibility and repeatability of the proposed segmentation, three operators (experienced radiologist, pulmonologist and image analyst) segmented multiple nodules (N = 266) from the NLST Controls cohort. The segmentation masks generated by the operators were compared pairwise using Dice Similarity Coefficient (DSC; **Figure B**). The 95% CI for the DSC between radiologist-pulmonologist, radiologist-image analyst and pulmonologist-image analyst was respectively 0.792-0.772, 0.785-0.804 and 0.835-0.857.

**1.2** **Nodule Surface characterization.** Nodule shape descriptors such as sphericity, flatness, elongation, spiculation and lobulation are accepted predictors of malignant nodules. We have developed quantitative methods to characterize these features in an automated and reproducible fashion. In addition to these common-place descriptors we also investigated the applicability of unbiased curvature-based shape descriptors. In this approach, the nodule surfaces are meshed, cleaned and smoothed; mean and Gaussian curvatures are computed at the mesh vertices. Based on the vertex-wise signs of the mean and Gaussian curvatures, the vertices are classified into surface features as peak, ridge, saddle ridge, flat, minimal, pit, valley and saddle valley. We showed in a pilot study that unsupervised stratification based on cumulative distribution of these surface features distinguishes benign and malignant nodules. Consequently we designed, implemented and integrated all the components of nodule surface analysis into a single monolithic software. (**Table A**) Due to this significant development effort, the processing time per nodule (excluding the nodule segmentation time) has now been dramatically reduced to < 1s.

| Tools used in Pilot Study | Components | Current Status |
| --- | --- | --- |
| ANALYZE | **Nodule Segmentation** | CANARY-PLUS |
| ANALYZE AVW | **Surface Extraction** | In-house monolithic software |
| ADMesh | **Surface Repair** |  |
| MATLAB | **Surface smoothing** |  |
| MeshLab | **Discrete Curvatures Estimation** |  |
| MATLAB | **Surface feature categorization** |  |
| CALIPER | **Unsupervised clustering** |  |
| ~ 30 minutes | **Time to process one nodule** | **< 1 second** |

**Table A**


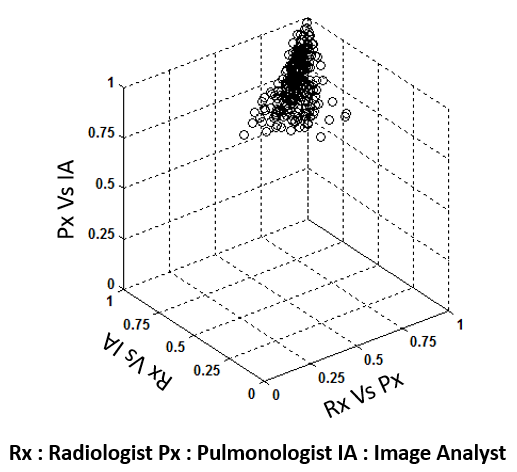


**Figure B**

S1.3 **Development of Score Indicative of Lesion/Lung Aggression/Abnormality (SILA).** Current literature suggests that no single quantitative metric exists to differentiate benign and malignant nodules. However, multivariate predictive models based on an ensemble of nodule texture, surround texture, nodule surface and other shape descriptors could improve the discriminability. To facilitate the multivariate analysis we investigated the possibility of replacing our previously developed nodule texture and surface categorization using unsupervised stratification into continuous variables that can be thresholded at multiple levels to provide, if needed, the necessary categorization. We developed SILA to map the nine nominal texture/surface exemplar distributions of the nodule onto a continuous scale. The nine nominal exemplar distributions can be ordinated in 362,880 (factorial 9) ways. To identify the unique ordination that correlates with the virulence/malignancy of the nodule, we used qualitative spatial reasoning and multi-dimensional scaling. Based on this, the nine texture exemplars arbitrarily labeled as V,I,B,G,Y,O,R,C, and P were ordinated as V-R-O-I-Y-P-B-G-C identical to that used to represent the distributions via the glyphs. (**Figure C**) Are A and D malignant versus benign nodules ??? The nine surface exemplars were ordinated as unknown-minimal surface-valley-flat-ridge-pit-saddle valley-saddle ridge-peak. SILA was computed as the Cramer-Von Mises Distance of the ordinated exemplar distributions. Using a similar strategy, the seven primal parenchymal exemplars (Normal, Ground Glass, Honeycombing, Reticular, {mild, moderate, severe} lower attenuation areas) were ordinated to compute the SILA for the parenchyma surrounding the nodule. **Figure D** shows the operator dependent variations in the SILA mappings for the texture and surface characterization. The 95% C.I for the maximum nodule-specific SILA differences across the 3 operators was 0.217 – 0.271 and 0.236 – 0.276 respectively for the texture and surface characterization.


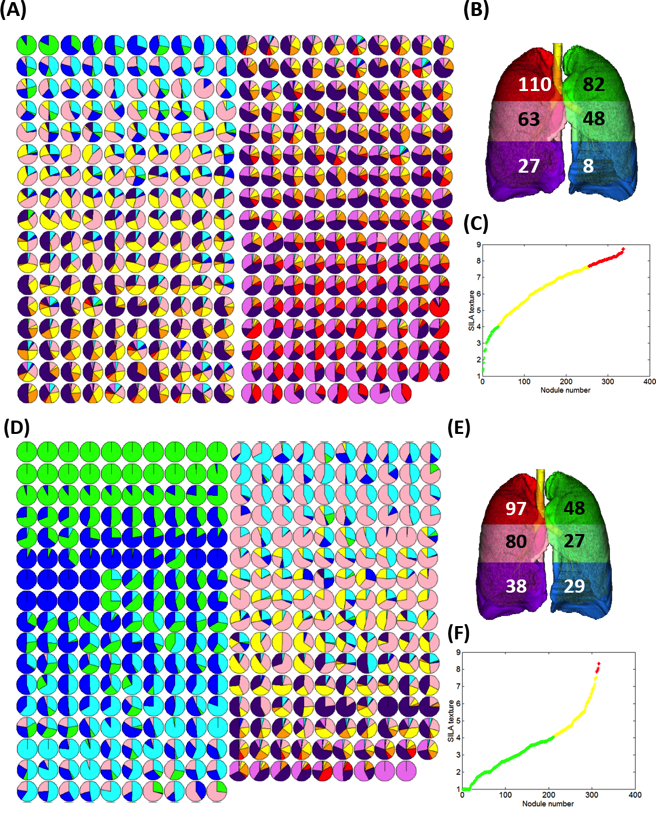


**Figure C**


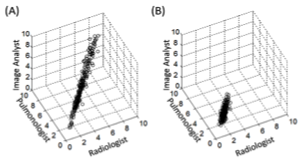


**Figure D**

2.1 **Quantitative metrics for the discrimination of benign and malignant lung nodules**. A comprehensive number of automatically computable, quantitative metrics were identified for the discrimination of benign and malignant lung nodules (Table B). These metrics are broadly categorized into

1. Metrics capturing the spatial **Location** of the nodule.
2. **Nodule Size**
3. Bulk metrics based on the global **Shape** descriptors of the nodule.
4. **Radiodensity** metrics based on the CT Hounsfield units within the nodule.
5. Nodule **Texture** metrics based on the texture exemplar distributions within the nodule.
6. **Texture nodule surrounding lung** metrics based on the parenchymal texture exemplar distributions within a region surrounding the nodule.
7. Metrics capturing the **nodule surface** descriptors.
8. Metrics capturing the **distribution of the nodule surface characteristics exemplars**.

The discriminability of benign and malignant nodules were analyzed using t-test and the normal distribution of the individual metric value was sassed using the quantile-quantile plot and the probability plot correlation coefficient (PPCC). In contradiction to the existing literature, our investigation revealed that skew and kurtosis of the voxel intensities within the nodule is not a discriminator of benign and malignant nodules. This could be attributed to the majority of current work focused on solid nodules.

Table B.

| **Metric Category** | **#** | **Metric** | **Description** | **Pval**  **95% CI for Benign /Malignant**  **PPCC for Benign/ Malignant** |
| --- | --- | --- | --- | --- |
|  | 1 | Volume (V) | Nodule volume in mm^3 | < 0.0001 254 – 435 / 2521 – 5716  0.57 / 0.45 |
|  | 2 | Surface Area (S) | Surface in mm^2. Contribution of each surface voxel is determined by the neighbors of that voxel. Additionally, the surfaces which intersect the edge of the volume are included in the measurements. | < 0.0001 289 – 399 / 2479 – 2299  0.71 / 0.61 |
|  | 3 | Sphericity (Sph) | $Sph= \frac{6*\surd\pi V}{S^{3/2}}$ | < 0.0001 0.57 – 0.63 / 0.48 – 0.53  0.94 / 0.97 |
|  | 4 | Sphere Fit Factor (SFF) | $SFF= \frac{S^{3}}{\pi\left( 2V/3 \right)^{2}}$ | 0.0008 4.63 – 5.92 / 6.3 – 8.18  0.82 / 0.77 |
|  | 5 | Radius (R) | Maximum radius if it were to be spherical nodule . $R= \left( \frac{3V}{4\pi} \right)^{1/3}$ | < 0.0001 3.42 – 3.77 / 7.1 – 7.9  0.88 / 0.89 |
|  | 6 | Minimum Enclosing Brick(MEB) | The x,y,z extents of the minimum enclosing brick around the nodule |  |
|  | 7 | MEB Angle | The orientation of the minimum enclosing brick around the nodule |  |
|  | 8 | Elongation (E) | $E=1.0- \frac{\varepsilon\_max}{\varepsilon\_mid}$  ε_max, ε_mid are the maximum and middle edge lengths of MEB | **0.45** -0.36 – -0.26 / -0.34 – -0.23  0.72 / 0.7 |
|  | 9 | Flatness (F) | $F=1.0- \frac{\varepsilon\_mid}{\varepsilon\_min}$  ε_min, ε_mid are the minimum and middle edge lengths of MEB | < 0.0001 -1.12 – -0.89 / 0.68 – -0.46  0.98 / 0.96 |
| **Intensity** | 10 | Mean (Avg) | average of the voxel intensities within the nodule | < 0.0001 -487 – -443 /-233 – -195 |
|  | 11 | Variance (Var) | variance the voxel intensities within the nodule | **0.06**  **-**  .66 / .35 |
|  | 12 | Skew (Skew) | skewness of the voxel intensities within the nodule  $Skew= \frac{\frac{\sum({x_{i}-Avg)}^{3}}{N}}{{Var}^{3/2}}$ | **0.55** -25 – -2.6 / -3.9 – -1.6  .95 / .42 |
|  | 13 | Kurtosis (Kur) | Kurtosis of the voxel intensities within the nodule  $Kur= \frac{\frac{\sum({x_{i}-Avg)}^{4}}{N}}{{Var}^{2}}$ | **0.24** 9.43 – 11.64 / -82.34 – 395.39  .68 / .17 |
|  | 14 | Entropy (En) | $En= -x_{i}*log2(x_{i})$ | < 0.0001 6.57 – 6.96 / 7.47 – 7.88  .68 / .69 |
| **Location** | 15 | Location (Loc) | The Left/Right (L/R), Upper/Middle/Lower (U/M/L), Peripheral / Central (P/C) region containing majority of the nodule voxels. The bar chart on the right shows the relative distribution of the benign (blue) and malignant (red) nodules across the 12 regions. | 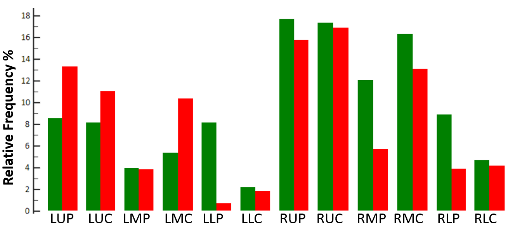PPCV: .97 / .96 |
|  | 16 | Offset from Carina | x,y,z- offsets of the nodule centroid to Carina |  |
|  | 17 | Offset from Hila | x,y,z- offsets of the nodule centroid to Hila |  |
|  | 18 | Offset from Pleura | x,y,z- offsets of the nodule centroid to Pleura |  |
| **Nodule Texture** | 19 | Exemplar Distribution | Distribution of the nine CANARY texture exemplars constituting the nodule |  |
|  | 20 | SILA | Score Indicative of Lesion Aggression for the nodule texture. | < 0.0001 54.42 – 62.81 / 116.88 – 124.58  0.98 / 0.97 |
|  | 21 | Risk Category | Risk Stratification group. The bar chart on the right shows the relative distribution of the risk groups for benign (green) and malignant (red) nodules. | 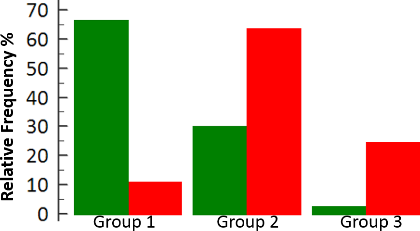PPCV: .8 / .87 |
| **Nodule Surround Texture** | 22 | Surround Distribution | Distribution of the 7 parenchymal exemplars (Normal, Honey comb, Reticular, Ground glass, mild low attenuation area (LAA , moderate LAA, severe LAA) within 10 mm around the nodule. Bar chart on the right shows the average % distribution of non-normal exemplars for the benign (green) and red (malignant) nodules | 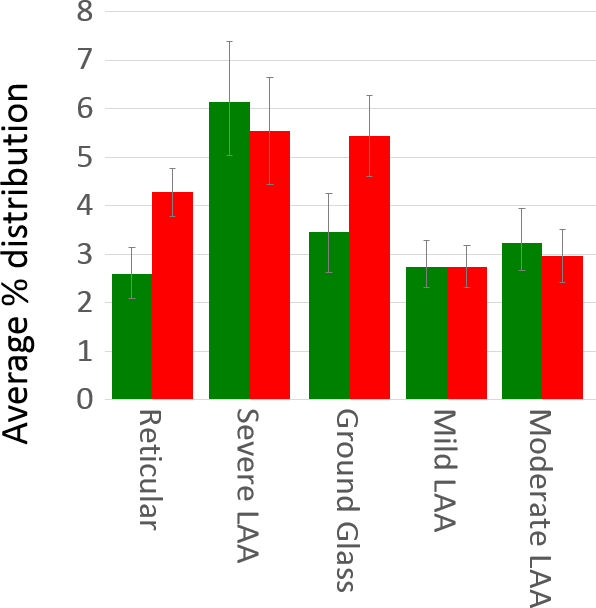 |
|  | 23 | Percentage Vessels | Percentage of Vessels within a 10 mm region around the nodule | < 0.0001 0.61 – 0.89 / 1.41 – 1.77  0.8 / 0.91 |
|  | 24 | Percentage Background | Percentage of non-lung voxels around the nodule | **0.928** 8.35 – 10.81 / 8.65 – 10.68  0.9 / 0.94 |
|  | 25 | SILA_Fib | SILA score for the distribution of honey comb, reticular and ground glass surrounding the nodule. | 0.001 24.89 – 29.95 / 30.62 – 34.48  0.96 / 0.99 |
|  | 26 | SILA_LAA | SILA score for the distribution of mild, moderate and severe LAA. | 0.05 30.5 – 34.88 / 33.62 – 37.2  0.98 / 0.99 |
| **Nodule Surface** | 27 | Number of Vertices NV | Number of vertices in the surface | < 0.0001 438 – 592 / 2262 – 3373 0.72 / 0.63 |
|  | 28 | Willmore Bending Ene-rgy (WEB) | Given the per-vertex principal curvatures *k_1_* and *k_2_,* $WEB= \int_{A} \left( k_{1}^{2}+k_{2}^{2} \right)dA$ | < 0.0001 401 – 560 / 1377 – 2279  0.72 / 0.57 |
|  | 29 | H: Min | Mean curvature (H) is an extrinsic curvature that arises from the mechanical folding of the surface.  $H= \frac{\left( k_{1}+k_{2} \right)}{2}$ | < 0.0001 -0.33– -0.23 / -1.05 – -0.90  0.89 / 0.97 |
|  | 30 | H: Max |  | < 0.0001 3.07 – 3.47 / 3.82 – 4.45  0.84 / 0.87 |
|  | 31 | H: -ve Avg |  | **0.2** -0.14 – -0.12 / -0.15 – -0.13  - / - |
|  | 32 | H : +ve Avg |  | < 0.0001 0.56 – 0.60 / 0.35 – 0.37  0.98 / 0.94 |
|  | 33 | H : -ve Skew |  | < 0.0001 -1.1 – -0.85 / -2.2 – -1.97   - / - |
|  | 34 | H: +ve Skew |  | < 0.0001 1.88 – 2.14 / 2.97 – 3.44  0.98 / 0.94 |
|  | 35 | G: Min | Gaussian Curvature (G)  $G=k_{1}*k_{2}$ | 0.0005 -0.96 – -0.78 / -1.21 – -1.01  0.86 / 0.88 |
|  | 36 | G: Max |  | 0.0004 10.27 – 14.93 / 17.41 – 26.62  0.6 / 0.64 |
|  | 37 | G: -ve Avg |  | **0.30** -93 – 30.21 / -0.06 – -0.055  0.16 / 0.86 |
|  | 38 | G: +ve Avg |  | < 0.0001 0.56 – 0.67 / 0.31 – 0.44  0.84 / 0.61 |
|  | 39 | G: -ve Skew |  | < 0.0001 -2.52 – -2.2 / -4.46 – -4.0   - / - |
|  | 40 | G: +ve Skew |  | < 0.0001 4.43 – 4.9 / 7.41 – 8.21  0.94 / 0.96 |
|  | 41 | Sharpness (S): Min | Sharpness at a vertex emphasizes regions where *k1* and *k2* are maximally different as in the crests and depths.  $S_{i}= \left( k_{1}^{2}- k_{2}^{2} \right)$ | < 0.0001 -0.8 – -0.64 / -1.6 – -1.4  0.95 / 0.98 |
|  | 42 | S: Max |  | < 0.0001 3.56 – 4.16 / 5.12 – 5.96  0.85 / 0.91 |
|  | 43 | S: Avg |  | < 0.0001 0.65 – 0.70 / 0.39 – 0.42  0.98 / 0.97 |
|  | 44 | S: Skew |  | < 0.0001 1.1 – 1.34 / 1.65 – 2.01  0.94 / 0.91 |
|  | 45 | Curvedness (C): Min | Curvedness captures information on less sharp folding thus revealing smaller bumps and ridges.  $C= \sqrt{\left( \frac{k_{1}^{2}+k_{2}^{2}}{2} \right)}$ | < 0.0001 0.03 – 0.04 / 0.003 – 0.005  0.88 / 0.87 |
|  | 46 | C: Max |  | < 0.0001 2.35 – 2.68 / 3.10 3.63  0.83 / 0.89 |
|  | 47 | C: Avg |  | < 0.0001 0.47 – 0.51 / 0.30 – 0.32  0.98 / 0.94 |
|  | 48 | C: Skew |  | < 0.0001 1.71 – 1.99 / 2.94 – 3.46  0.90 / 0.87 |
|  | 49 | Shape Index (SI): Min | $SI= \frac{2}{\pi} \tan^{-1} \left( \frac{k_{1}+k_{2}}{k_{2}-k_{1}} \right)$ | < 0.0001 -0.62 – -0.48 / -0.1 – -0.96  0.81 / 0.33 |
|  | 50 | SI: Max |  | < 0.0001 0.975 – 0.978 / 0.982 – 0.984  0.91 / 0.94 |
|  | 51 | SI: Avg |  | < 0.0001 0.54 – 0.56 / 0.28 – 0.31  0.97 / 0.99 |
|  | 52 | SI: Skew |  | 0.5 -1.87 – -1.56 / -1.75 – -1.56 |
|  | 53 | Intrinsic Curvature Index (ICI) | ICI counts the number of regions with undulations or saliencies on the surface.  $ICI= \frac{1}{4\pi} \iint\left\vert k_{min} k_{max} \right\vert dA$  where  $k_{min}= H- \sqrt{H^{2}-G}$  $k_{max}= H+ \sqrt{H^{2}-G}$ | < 0.001 13.33 – 18.08 / 34.94 – 64.15  0.72 / 0.81 |
|  | 54 | Extrinsic Curvature Index (ECI) | ECI counts the number and length (with respect to the diameter) cracks and gaps on the surface.  $ECI= \frac{1}{4\pi} \iint\left\vert k_{mzx} \right\vert\left( \left\vert k_{max} \right\vert- \left\vert k_{min} \right\vert\right) dA$ | < 0.0001 33.13 – 45.7 / 100.2 – 167.8  0.71 / 0.82 |
| **Morpheme** | 55 | Morpheme Distribution | The distribution of the nine morphometric exemplars of the nodule surface. The plot on the right shows the average cumulative distribution between the benign (green) and malignant (red) nodules. | 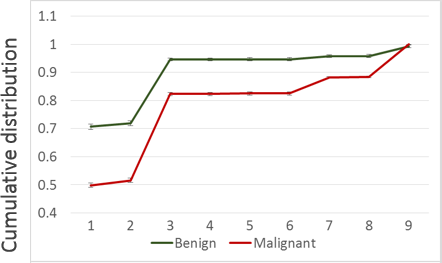 |
|  | 56 | SILA Morpheme | Score Indicative of Lesion Abnormality based on the morpheme distribution. The plot on the right shows the distribution of SILA morpheme for benign and malignant nodules | 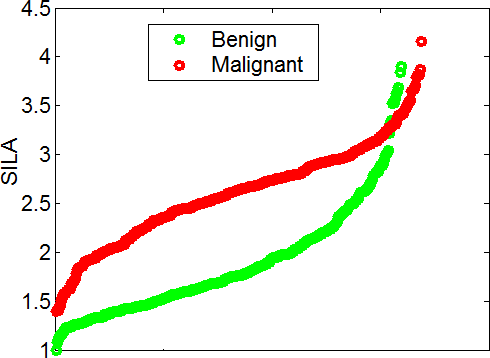< 0.0001 1.81 – 1.94 / 2.55 – 2.66  0.95 / 0.99 |
|  | 57 | Morpheme Curvedness (MC): Avg | Morpheme-wise average curvedness of the surface. The table to the right shows the pvals and 95% CI for the benign and malignant nodules. Sig = Significant ( < 0.0001); NS = not significant | \| **Id** \| **p** \| **95% CI** \| \| --- \| --- \| --- \| \| 1 \| Sig \| 0.52 – 0.56/ 0.39 – 0.41 \| \| 2 \| **NS** \| 0.007– 0.03/0.008– 0.17 \| \| 3 \| Sig \| 0.4 – 0.44 / 0.26 – 0.28 \| \| 4 \| - \|  \| \| 5 \| - \|  \| \| 6 \| - \|  \| \| 7 \| **NS** \| 0.21 – 0.26 / 0.2 – 0.23 \| \| 8 \| **NS** \| 0.01 – 0.04 / 0.01 – 0.02 \| \| 9 \| Sig \| 0.2 – 0.23 / 0.15 – 0.16 \| |
|  | 58 | MC: Skew | Morpheme-wise skew of curvedness of the surface. The table to the right shows the pvals and 95% CI for the benign and malignant nodules | \| **Id** \| **p** \| **95% CI** \| \| --- \| --- \| --- \| \| 1 \| Sig \| 1.51 – 1.73 / 2.38 – 2.76 \| \| 2 \| **0.02** \| 2.6 – 3.8 / 3.75 – 4.59 \| \| 3 \| Sig \| 1.04 – 1.31 / 2.15 – 2.54 \| \| 4 \| - \|  \| \| 5 \| **0.32** \| 0.46 – 2.33 / 1.45 – 8.7 \| \| 6 \| - \|  \| \| 7 \| Sig \| 0.33 – 0.57 / 1.21 – 1.41 \| \| 8 \| **0.07** \| 0.21 – 1.7 / 1.6 – 2.4 \| \| 9 \| Sig \| 0.49 – 0.71 / 1.32 – 1.52 \| |
|  | 59 | Local SILA : Avg | 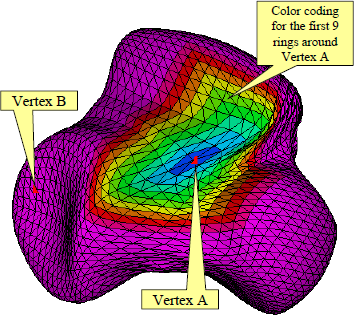Local morpheme SILA was computed by finding 2-ring neighbors around each vertex. The figure below shows the color coded 9-ring neighbor around a sample vertex for a representative nodule.  The average and skew of the local SILA distribution was computed. | < 0.0001 14.38 – 16.32 / 26.74 – 28.64  0.95 / 0.99 |
|  | 60 | Local SILA: Skew |  | < 0.0001 0.41 – 0.56 / 0.66 – 0.75  0.99 / 0.99 |
|  | 61 | Local SILA: Histogram | Figure to the right shows the average cumulative distribution of the local morpheme SILA for the benign and malignant nodules | 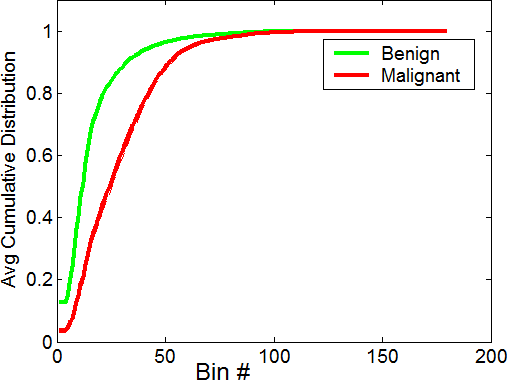 |
